# Sample size calculation for multicentre efficacy trials of blood-stage malaria antigens

**DOI:** 10.1186/1475-2875-12-253

**Published:** 2013-07-19

**Authors:** Samuel Bosomprah

**Affiliations:** 1 Department of Biostatistics, School of Public Health, University of Ghana, Legon, Accra, Ghana

## Abstract

**Background:**

Sample size has increasingly become a prerequisite for grant approval. Study size calculations for multicentre trials are more complicated because these sites present different assumptions on incidence of disease expected in the control group; this then changes the mechanism of sample size determination. This paper suggested an alternative approach to estimating study size in multicentre vaccine efficacy trials.

**Methods:**

The approach suggested in this paper was to determine the expected number of events for a given sample size under set of different assumptions. The power was then calculated given the expected number of events under the set of assumptions so as to assess the sensitivity of the sample size. The approach was then illustrated assuming a malaria vaccine efficacy trial planned in four centres.

**Results:**

The approach showed that by assuming 30% cumulative incidence of malaria in three of the centres and 10% cumulative incidence in the other centre, a sample size of 460 children in each centre (total 1,840) corresponding to a total of 339 events gives 90% power to detect vaccine efficacy of 30% at 5% level of significance, allowing for 15% loss to follow-up. However, if the incidence is lower than anticipated or a centre drops out altogether the power will be low. But this would not have much effect if it were a low incidence centre. Rather, it might have major effect if it were a high incidence centre.

**Discussion:**

Decision on recruitment depends on whether separate estimates of efficacy in each transmission level are reasonable. If not, equal numbers can be recruited, which then gives safety data for each site and overall efficacy. Recruiting all or most subjects in the highest transmission site can minimize sample size but may be better to spread the risk due to uncertainty about incidence due to year to year variation and also the possibility of a site dropping due to political or other unforeseen problems.

**Conclusion:**

The approach demonstrated the potential of estimating the expected number of events required to give a specified power for multicentre efficacy trails of blood stage malaria antigens.

## Background

Planning vaccine efficacy trials requires a major decision on the number of subjects to enrol on the study in order to give reasonable statistical power to detect a true effect. Several approaches – confidence interval and power – exist for calculating sample size depending on the aim of the study. If the aim is to obtain estimate of an intervention with a specified level of precision then one can specify the desired width of the confidence interval and work out the sample size that achieves that objective. The power approach aims to estimate a sample size to achieve a specified power and this is the approach emphasized in this paper. The basic principle is to quantify the primary objective of the study in terms of certain statistical parameters. Specifically, the statistical considerations are to state a null hypothesis with its associated type I error rate and an alternative hypothesis with its associated statistical power; also, the test statistic that distinguished between the two hypotheses [1,2]. Having specified these parameters, an exercise is then performed to determine the number of participants required to achieve the stated type I error rate and the power simultaneously. For test statistic that has standard distributional properties, one can easily apply the standard formula to estimate the sample size. However, controlled trials often deviate from standard assumptions so that the test statistic becomes more complicated. In such complicated studies, one can approach the sample size estimation in three ways. First, one can use the standard formula to approximate the study size over a possible range of parameters given a set of ideal assumptions, such as no loss to follow-up, independence of events, full compliance, among others. This gives an idea about the resources needed in the study. Second, having identified the likely deviation from the assumption, one can adjust the study size accordingly. Finally, if the trial includes highly specialized features, one can consider simulation to select a more appropriate study size [3,4]. The exercise of sample size calculation can be iterative. For instance, one can extend the follow-up time to reduce the sample size. One can also change the inclusion criteria to increase event rates. One can even select study sites that have a good history of retention in order to reduce loss to follow-up. This systematic approach is aimed at producing a reasonable sample size for the study because a sample size too small can lead to low statistical power, whereas too large a sample can be a waste of time and resources and can also be unethical.

In trials with binary outcomes or time to event outcomes, the word “small” refers not to the number of patients studied but rather to the number of events observed. For instance, a trial of 3,000 children on placebo and 3,000 on a new vaccine being followed for 12 months to study the effect of the new vaccine in preventing clinical malaria can be considered as “small” in the terminology of controlled trials, if it suggests that only about 20 events are expected to occur in the control group. The 99% or so of children who do not experience clinical malaria provide essentially no information about the effect of the vaccine. Therefore, an alternative approach that estimates the expected number of events in the control group required to give a specified power forms part of this paper. The sample size in a controlled trial cannot be arbitrarily large. The total number of participants potentially available, the budget, and the amount of time available can influence the number of participants to be included in a trial. The sample size of a trial must be adequate to allow a reasonable chance of answering the research question but not so large that continuing randomization will lead to ethical discomfort. Where a larger number of participants are anticipated in different epidemiological settings with a wider range of population groups, one strategy is to carry out the study in more than one centre - multicentre design. This is particularly useful where the number of potential participants available in a study centre is limited. It ensures the ability to compare results among centres and increases the generalizability of the results. Such trials have been conducted during the past decade [5,6]. The design and analysis of these trials are not straightforward compared with single-centre trials. This is because these centres present different assumptions on incidence of disease expected in the control group, which then changes the mechanism of sample size determination. In vaccine trials, the primary objective is often to compare incidence rate or hazard rate of some disease, say, malaria in the intervention group with that in the control group and so this paper focused on comparison of incidence rates in two groups.

## Methods

Donner reviewed approaches to sample size estimation in the design of clinical trials [7]. Sample size formulae depend on the type of primary outcome being investigated in the study and are guided by both statistical and resource considerations.

Smith and Morrow [2] showed that for single-centre trial, the person-years at risk, y, of observation in each group is given by:

(1) $$y=\frac{{\left(z_{\alpha /2}+z_{\beta }\right)}^{2}\left(\lambda_{c}+\lambda_{v}\right)}{{\left(\lambda_{c}-\lambda_{v}\right)}^{2}}\;$$

Where *z*_*α*/2_ is the percentage of the standard normal distribution corresponding to the required two-sided significant level (for example, if significance level =5%, *z*_*α*/2_ =1.96); z_*β*_ is one-sided percentage point of the standard normal distribution corresponding to 100% - the power (for example, if power = 90%, (100% - power) =10% and *z*_*β*_ =1.28); *λ*_*c*_ is the incidence rate in the control group and *λ*_*v*_ is the incidence rate in the vaccine group. By definition, rate is the number of new cases divided by total person-time at risk. Therefore, the number of events in each group is given by the product of rate, *λ*, and person-time at risk, *y.* So, if it is assumed that the person-years at risk is the same in both group the total expected number of events, *E(n),* is given by:

(2) $$E\left(n\right)=\left(\lambda_{c}+\lambda_{v}\right)y$$

the sample size necessary to expect *E(n)* events can be derived from equations (1) and (2) using:

(3) $$N=y*2+E\left(n\right)*M$$

where E(n)*M is the adjustment factor for risk-free period after anti-malarial treatment. That is, M is the number of weeks the child is assumed not at risk, expressed in year, after each treatment. For example, if it is assumed that the child is not at risk for three weeks after anti-malarial treatment then M=3/52.

The incidence rate in the control population *λ*_*c*_ could be derived from the cumulative incidence, *r*:

(4) $$\mathrm{Risk}\;\mathrm{up}\;\mathrm{to}\;\mathrm{time},t=r=1-e^{-\mathrm{\lambda t}}$$

Re-arranging eqn (4) gives

(5) $$\lambda_{c}=-\ln\left(1-r\right)/t$$

For multicentre trials the formula becomes more complicated because one needs to work out the expected number of events in each centre, assuming a specified sample size, to give a reasonable power. The total expected number of events, *E*(*n*), in terms of the relative rate, *θ,* and the sample size, *N,* for an expected incidence rate in the control group, *λ*_*c*_, assuming a fraction loss to follow up, *L*, is given by:

(6) $$E\left(n\right)=\left[\lambda_{c}+1-\exp\left(-\left(-\ln\left(1-\lambda_{c}\right)\theta \right)\right)\right]\frac{N}{2}*\left(1-L\right)$$

The power for a given expected number of events required in the control group, *E*_*c*_(=*E*(*n*)/1 + *θ*), for a specific relative rate, *θ*, could be derived from the formula:

(7) $$z_{\beta }=\sqrt{\frac{E_{c}{\left(1-\theta \right)}^{2}}{1+\theta }}-z_{\alpha /2}$$

The power is given by returning the standard normal cumulative distribution of *z*_*β*_

### Illustrative case studies

Assume a sample size requirement for a malaria vaccine efficacy trial planned in four centres: A, B, C and D. The primary objective of the trial was to assess the efficacy of a candidate vaccine against *Plasmodium falciparum* clinical malaria episodes in children aged 12–60 months at first vaccination over a six-month surveillance period, starting from the day of the third dose of vaccination. It was proposed that the primary analysis would be done as soon after six months of follow-up had elapsed if at least 330 children had had an episode of malaria. In case the target number is not reached by 12 months, analysis will be carried out at 12 months. Table 1 shows the sample size corresponding to total expected number of events under different scenarios. If 30% cumulative incidence of malaria in three of the centres (B, C, and D) and 10% cumulative incidence in centre A is assumed, then a sample size of 460 children in each centre (total 1,840) corresponding to a total of 339 events gives 90% power to detect vaccine efficacy of 30% at 5% level of significance, allowing for 15% loss to follow-up (Tables 1 and 2 - Scenario 1). If the incidence is lower than anticipated or a centre drops out altogether the power will be low. But this would not have much effect if it were a low incidence centre. Rather, it might have major effect if it were a high incidence centre. If incidence in centre A is very low, say 5%, rather than the 10% assumed as in Scenario 2, the power will be 89% to detect vaccine efficacy of 30%. If incidence is very low in both centres, A and B, say 5% as in Scenario 3, the power will be 78% to detect vaccine efficacy of 30%. If all the sites have incidence reduced to half, the power will be 63% to detect vaccine efficacy of 30%. If centre D drops out altogether the power will be 78% to detect vaccine efficacy of 30%. If the sample size is lower than anticipated due to, say, resource constraint, the expected number of events will be lower and the power will be less. If only 70% of the sample size anticipated can be recruited under the same assumption of incidence as in Scenario 1, the expected number of events will be reduced from 339 to 237 and the power will be 78% to detect vaccine efficacy of 30%.

**Table 1:** **Sample size in each site to give the required expected number of events under different scenarios using *eqn (***6***)***

**Centres**	**% malaria after six months**	**Sample size (all the required number can be recruited)**	**No of events [*eqn(***6**)]**	**Sample size (only 70% can be recruited)**	**No of events [*eqn(***6***)*]**
**Scenario 1: Sample size of 460 in each site with the best estimate of incidence rates**					
A	0.1	460	33	322	23
B	0.3	460	102	322	71
C	0.3	460	102	322	71
D	0.3	460	102	322	71
Total		1,840	339	1,288	237
**Scenario 2: Very low incidence in centre A**					
A	0.05	460	17	322	12
B	0.3	460	102	322	71
C	0.3	460	102	322	71
D	0.3	460	102	322	71
Total		1,840	322	1,288	226
**Scenario 3: Very low incidence in both centres A and B**					
A	0.05	460	17	322	12
B	0.05	460	17	322	12
C	0.3	460	102	322	71
D	0.3	460	102	322	71
Total		1,840	237	1,288	166
**Scenario 4: All sites have incidence reduced to half**					
A	0.05	460	17	322	12
B	0.15	460	50	322	35
C	0.15	460	50	322	35
D	0.15	460	50	322	35
Total		1,840	168	1,288	117
**Scenario 5: Centre D drops out of the study**					
A	0.1	460	33	322	23
B	0.3	460	102	322	71
C	0.3	460	102	322	71
Total		1,380	237	966	166

**Table 2:** **The power for expected number of events required in the control group for a specific relative rate, θ, for the different scenarios as in Table**1**using *eqn (***7***)***

**Scenarios**	**Sample size**	**E(n)**	**Alpha**	**z**_**α/2**_	**θ**	**E**_**c**_	**z**_**β**_	**Power**
Scenario 1	1,840	339	0.05	1.96	0.7	199.412	1.2892	0.90
	1,288	237	0.05	1.96	0.7	139.412	0.75677	0.78
Scenario 2	1,840	322	0.05	1.96	0.7	189.412	1.20669	0.89
	1,288	226	0.05	1.96	0.7	132.941	0.69297	0.76
Scenario 3	1,840	237	0.05	1.96	0.7	139.412	0.75677	0.78
	1,288	166	0.05	1.96	0.7	97.6471	0.3137	0.62
Scenario 4	1,840	168	0.05	1.96	0.7	98.8235	0.32736	0.63
	1,288	117	0.05	1.96	0.7	68.8235	-0.05114	0.48
Scenario 5	1,380	237	0.05	1.96	0.7	139.412	0.75677	0.78
	966	166	0.05	1.96	0.7	97.6471	0.3137	0.62

## Discussion

Sample size has increasingly become a prerequisite for grant approval. This paper suggested an alternative approach to estimating study size in multicentre vaccine efficacy trials. Study size calculations for multicentre trials are more complicated because these sites present different assumptions on incidence of disease expected in the control group; this then changes the mechanism of sample size determination. The approach suggested in this paper is to determine the expected number of events for a given sample size under set of different assumptions. The power is then calculated given the expected number of events under the set of assumptions so as to assess the sensitivity of the sample size. Even though investigators often spend much time on the number of participants to enrol on trial, the sample size is not the only factor that influences the power of a trial. The total number of primary outcome experienced by the population is a critical factor because a large sample size with low event rate in the population can lead to low power. Therefore, in designing a trial it is important to consider how the estimated number of primary outcome can be realised. One strategy to ensure enough number of events is to select a high-risk group for the trial. The age of participants might help in identifying this group. Although oversampling a high-risk group might give more cases, it may make it more difficult to generalize the results to the general population. Another strategy is to extend the duration of follow-up, but this might not be useful in areas of low transmission. It may be decided to evaluate vaccines in low transmission settings but the sample size would need to be very high. It may not be possible to power the study to be able to test for interactions. Increasingly, it may be difficult to measure efficacy against severe disease due to low incidence. Site selection should consider balance of need to represent different transmission levels, and need to have optimum power for given sample size. Decision depends on whether separate estimates of efficacy in each transmission level are reasonable. If not, equal numbers can be recruited, which then gives safety data for each site and overall efficacy. Recruiting all or most subjects in the highest transmission site can minimize sample size but may be better to spread the risk due to uncertainty about incidence due to year-to-year variation, and also the possibility of a site dropping due to political or other unforeseen problems.

## Competing interests

The author declares that he has no competing interests.
